# Atypical retinal pigment epithelial defects with retained photoreceptor layers: a so far disregarded finding in age related macular degeneration

**DOI:** 10.1186/s12886-017-0452-0

**Published:** 2017-05-15

**Authors:** Helena Giannakaki-Zimmermann, Giuseppe Querques, Inger Christine Munch, Daraius Shroff, David Sarraf, Xuejing Chen, Eduardo Cunha-Souza, Sarah Mrejen, Vittorio Capuano, Murilo W. Rodrigues, Charu Gupta, Andreas Ebneter, Martin S. Zinkernagel, Marion R. Munk

**Affiliations:** 1 Department of Ophthalmology and Department of Clinical Research, Inselspital, Bern University Hospital, University of Bern, Bern, Switzerland; 2 Bern Photographic Reading Center, Inselspital, University Clinic Bern, Bern, Switzerland; 3 Department of Ophthalmology, University Vita-Salute, IRCCS Ospedale San Raffaele, Milan, Italy; 4 Department of Ophthalmology, University Paris Est, CHI, Creteil, France; 5 Department of Ophthalmology, Zealand University Hospital, University of Copenhagen, Copenhagen, Denmark; 6 Shroff Eye Center, New Delhi, India; 7 Jules Stein Eye Institute, UCLA, Los Angeles, CA USA; 8 Greater LA VA Healthcare Center, Los Angeles, CA USA; 9 University of Sao Paulo, Sao Paulo, Brazil; 10 Faculdade de Medicina de Ribeirão Preto, Ribeirão Preto, Brazil; 11 Vitreous Retina Macula Consultants of New York, New York, USA; 12 Department of Ophthalmology, Northwestern University, Feinberg School of Medicine, Chicago, IL USA

**Keywords:** RPE tear, Geographic atrophy, Age-related macular degeneration, RPE-aperture, Photoreceptor

## Abstract

**Background:**

To report patients with age-related macular degeneration and atypical central retinal pigment epithelium (RPE) defects not attributable to geographic atrophy (GA) or RPE-tears with overlying preserved photoreceptor layers.

**Methods:**

Multimodal imaging case-series evaluating the course of atypical RPE- defects in patients with AMD using Color fundus images, Optical coherence tomography (OCT), OCT-Angiography, fundus autofluorescence (FAF) and fluorescein-angiography (FA).

**Results:**

Ten patients were identified. Three patients had a prior RPE-rip and were excluded. Seven patients with a mean follow-up period of 47 ± 38 months after the occurrence of the RPE-defect were included (age range 71–87 years). Mean distance Best corrected visual acuity (BCVA) at initial presentation was 0.36 ± 0.29logMAR and at last follow-up visit 0.51 ± 0.43logMAR. Patients presented with clinically apparent GA on funduscopy and FAF, but preserved photoreceptor layers on optical coherence tomography (OCT). On FA there was early hyperfluorescence and late pooling visible. Over time, migration of RPE/drusenoid material right above the Bruch’s membrane with concomitant decrease of hypoautofluorescence was detectable in 4 cases. An enlargement of the RPE-defect was apparent in the remaining 3 cases. The majority (*n* = 4) showed a drusenoid pigment epithelium detachment (PED) preceding the lesion.

**Conclusions:**

Beside GA and characteristic RPE-tears, another atypical form of RPE-defect with overlying preserved photoreceptor layers are found in AMD. This so far disregarded subgroup of patients present with reasonable visual function and long-term survival of photoreceptors layers. Repair mechanisms such as ingrowth of RPE/drusenoid material and persistent subretinal fluid (SRF), but also a RPE-independent visual cycle for cone photopigment within the neurosensory retina may contribute to their favorable course.

## Background

Classic retinal pigment epithelium (RPE) tears in age-related macular degeneration (AMD) usually occur in patients with a fibrovascular pigment epithelium detachment. In respective cases a RPE tear may occur spontaneously, after administration of intravitreal anti- VEGF, or after photodynamic therapy [1–4]. These RPE-tears result in poor visual function and loss of photoreceptors with the development of fibrous tissue and enlargement of the RPE defects [5, 6]. Beside the description of these characteristic RPE-tears, a few case reports of less characteristic, central RPE-defects with neurosensory retinal elevation and subretinal fluid overlying the PED exist in literature [7–10]. Cases with such atypical RPE-defects had AMD or suspected central serous chorioretinopathy (CSC) without definite evidence of a CNV-membrane and maintained reasonable visual function over a longer period of time [7–10].

Subretinal migration of RPE/drusenoid material was found in some of these cases, resembling a possible repair mechanism [8, 9, 11–13]. Most of respective cases were described before the OCT era, and the preservation of the photoreceptors overlying the area of RPE-defects was therefore only presumed based on the maintenance of reasonable visual function.

A detailed, coherent description of such cases with a long term follow up using multimodal imaging is still missing. The aim of this study is to describe patients presenting with atypical, central RPE-defects not attributable to classic RPE-tears or geographic atrophy (GA) that retain intact photoreceptor layers and discuss possible pathomechanism.

## Methods

### Patient selection and setting

This retrospective, observational case series collected cases from tertiary referring institutions: 1. Inselspital, University Hospital Bern, Bern, Switzerland, 2. Retina clinic, University of Sao Paulo, Brazil, 3. Stein Eye Institute, Los Angeles, USA, 4. Zealand University Hospital, Denmark, 5. Shroff Eye Center, New Delhi, India, 6. Vitreous Retina Macula Consultants of New York, USA, 7. University Paris Est, CHI, Creteil, France, and 8. University Vita-Salute, IRCCS Ospedale San Raffaele, Milan, Italy.

Patients were included if they had been diagnosed with AMD and presented with a center-involving RPE-defect with intact overlying photoreceptor layers and a minimum follow up of 6 months. Spectral domain optic coherence tomography (SD-OCT) had to reveal a center involving RPE-defect and a retained external limiting membrane (ELM) and ellipsoid zone (EZ) overlying the defect. On funduscopy, clinically apparent central geographic atrophy (GA) had to be present. Patients with evidence of rippling/retracted RPE at the margins of the lesion and with a fibrovascular PED with/without a history of anti-VEGF administration preceding the RPE-defect were excluded. Demographic and clinical data were collected and image analysis included color fundus and red-free photography, SD-OCT (Heidelberg Engineering, Germany and Zeiss, Zeiss AG, Germany), near-infrared reflectance, blue (488 nm) fundus autofluorescence (FAF) and fluorescein angiography (FA). Whenever available NIR (787 nm)-FAF and OCT-angiography (Heidelberg Engineering and Nidek) were investigated as well. Data are reported in mean, standard deviation (mean ± SD), ranges and frequencies.

## Results

**Fig. 1:**
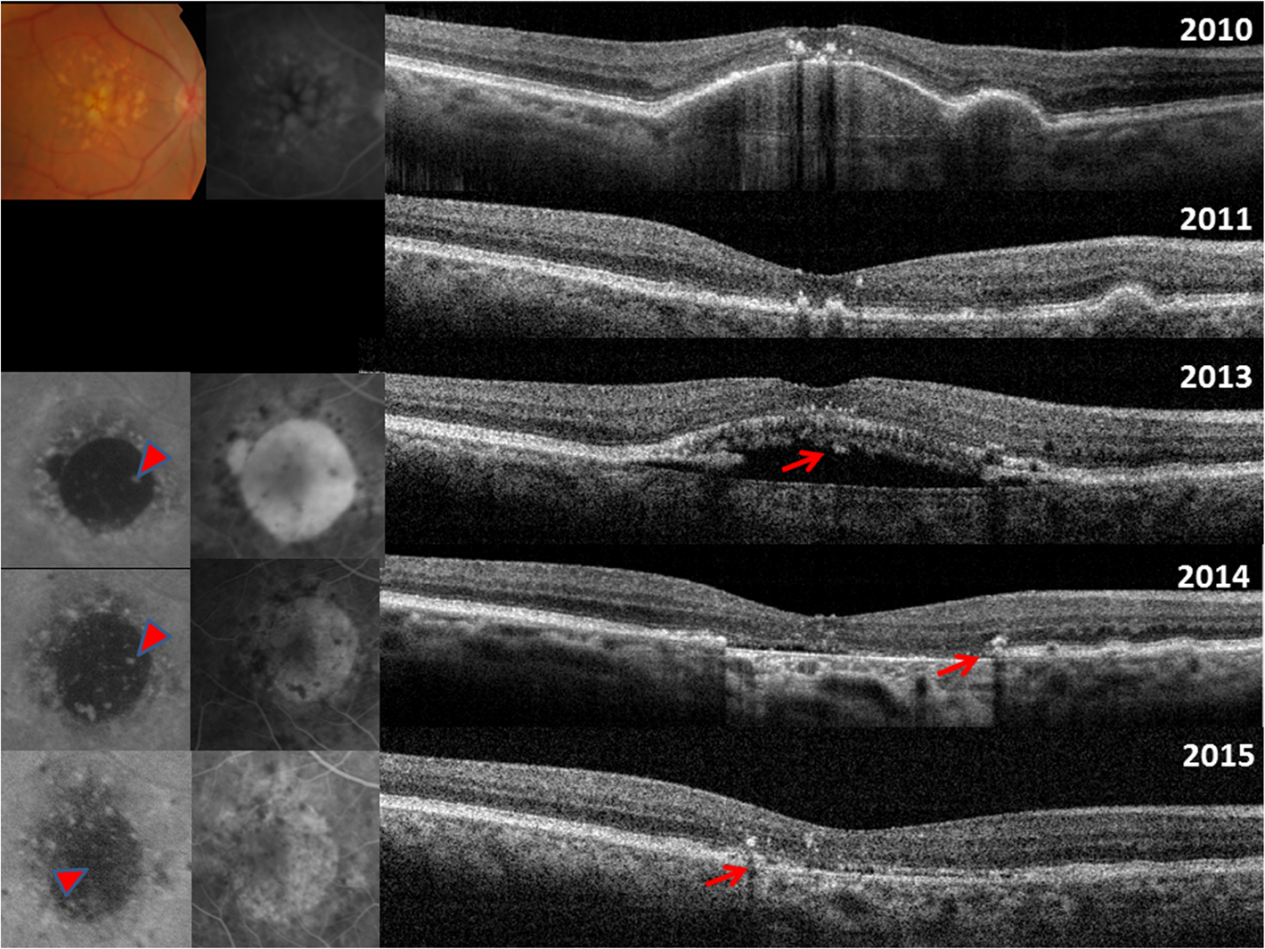
*Top*: 2010: Initial presentation with drusenoid pigment epithelium detachment (PED). 2011: spontaneous resolution of PED. 2013: Spontaneous RPE aperture. The OCT reveals an RPE defect with SRF. The overlying photoreceptors are intact. Fundus autofluorescence (FAF) and fluorescein angiography (FA) shows hypoautofluorescence and pooling corresponding to RPE defect, respectively. 2014 Resolution of SRF after anti-VEGF treatment. Intact ellipsoid zone and ELM directly attached to the Bruch’s membrane. Enhanced choroidal signaling due to the absence of RPE is evident. Some ingrowth of iso-autofluorescent material is already notable on FAF (*red, bold arrow*). Also SD-OCT reveal some hyperreflective material (*thin, red arrow*) 2015 Ingrowth and regeneration of RPE/drusenoid material with consecutive decrease of enhanced choroidal signaling (*thin, red arrow*), hypoautofluorescence (*red, bold arrow*) and window defect Red (*thin and bold*) arrows denote hyperreflective and hyperFAF material, suggestive of potential ingrowth of drusenoid/RPE material

**Fig. 2:**
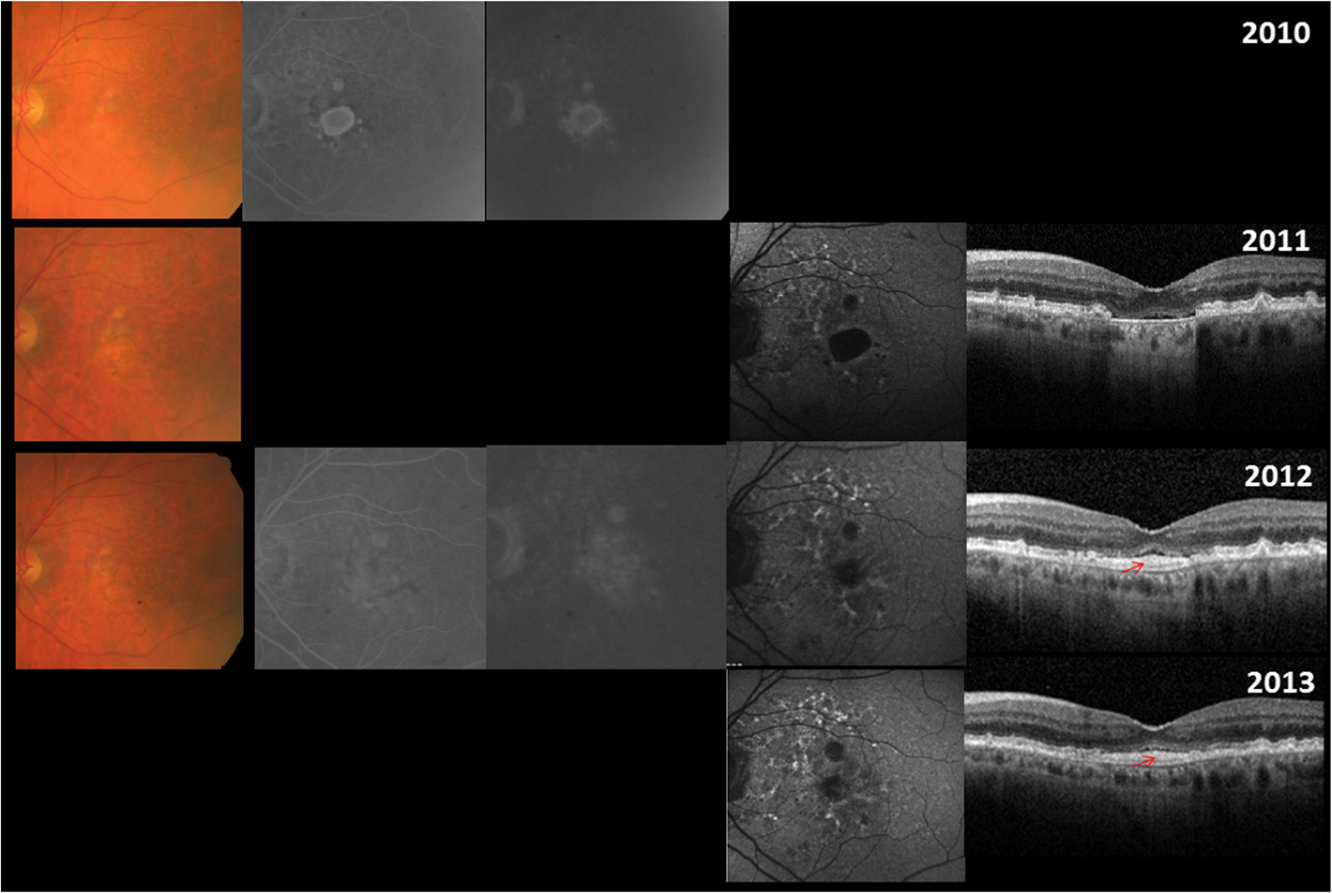
2010: A circumscribed RPE atrophy is noted on color fundus with corresponding window defects visible on FA. 2011: The now performed FAF and SD-OCT highlights the loss of RPE with corresponding hypo autofluorescence on FAF and enhanced choroidal signaling on SD-OCT. The ELM, ellipsoid zone and the interdigitation zone are attached to the Bruch’s membrane. 2012 and 2013: Decrease in window defect on FA, hypoautofluorescence on FAF and enhanced choroidal signaling on SD-OCT is noted. Ingrowth of RPE/drusenoid material (*red, thin arrow*) is evident overlaid by PR layers

**Fig. 3:**
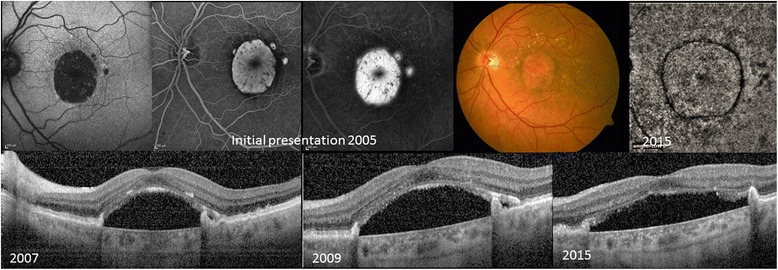
Initial presentation 2005 shows a large RPE atrophy on color fundus with corresponding hypoautofluorescence on FAF. A window defect with evidence of pooling within the subretinal space is visible on FA without a definite evidence of a CNV. 2007: Follow up images 2007 reveal a central RPE defect, SRF and a preserved photoreceptor (PR) layer. Obvious shedding of the PR-outer segments is evident. 2009: The RPE defect has increased in size, the SRF has persisted despite anti-VEGF therapy. The PR are present. 2015 Increase of the RPE defect, the overlying PR are still present but the lengths are reduced. The OCT angiography of the choriocapillaries confirms the absence of a CNV

**Fig. 4:**
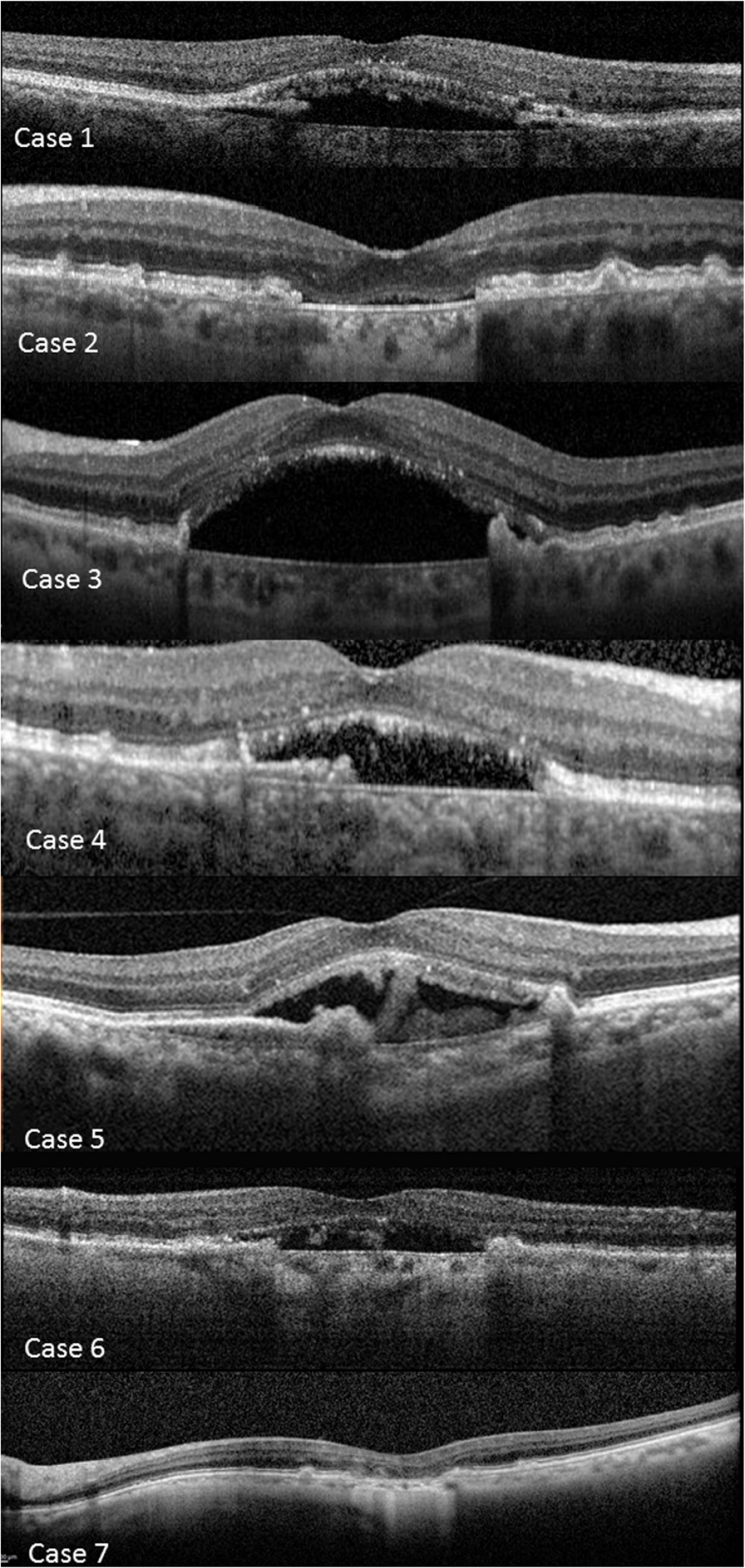
Depicts the presentation on OCT of each included patient

Ten patients were identified; three had a prior RPE rip and were excluded from the study. Thus, seven eyes from 7 patients (5 females) met the inclusion criteria. Demographic characteristics, ocular manifestation and administered treatment of each patient can be found in Table 1. Age ranged from 71–87 years. Mean distance BCVA at initial presentation was 0.36 ± 0.29logMAR and at last follow-up visit 0.51 ± 0.43logMAR (Table 1). Mean follow-up period was 47 ± 38 months. Patients presented with clinically apparent central geographic atrophy on funduscopy. FAF-imaging showed corresponding hypoautofluorence with stippled hyperautofluorescent areas scattered within the hypoautofluorescent lesion (Figs. 1, 2 and 3). SD-OCT revealed preserved ELM and EZ overlying the center involving RPE-defect throughout the entire observation period (Figs. 1, 2, 3 and 4). But while the ELM remained unchanged, the EZ decreased in thickness over time. The majority of eyes (*n* = 5) had subretinal fluid (SRF) overlying the RPE-defect (Figs. 1 and 3). Intact ELM and EZ directly adjacent to the Bruch’s membrane were found in the remaining 2 eyes (Fig. 2). On fluorescein-angiography there was an early hyperfluorescence corresponding to the RPE-defect and pooling in the late frames (Fig. 3). None had a definite evidence of exudative AMD. The absence of a CNV was confirmed by OCT-A in 3 cases (Fig. 3). None of the cases showed retracted RPE (Figs. 1, 2, 3 and 4).

**Table 1:** Clinical presentation and ocular characteristics of included patients

#	Eye	Lesion prior to RPE defect	F/u (mo)	BL BCVA	Final BCVA	Treatment after RPE defect	SRF duration (month)	OCT-A	HypoFAF area F/u	Mi-gration	RPE defect (OCT)	Presentation Fellow eye
1	OD	drusenoid PED	36	20/20	20/20	3× bevacizumab	12	-	↓	+	↓	Drusen, RPD
2	OS	N.A.	60	20/160	20/200	none	0	-	↓	+	↓	Occult CNV
3	OS	N.A.	120	20/50	20/200	2× retaane, 37× ranibizumab	persistent	No NV network	↑	-	↑	Drusenoid PED, IRC, atrophy
4	OD	drusenoid PED	60	20/40	20/125	32× ranibizumab	persistent	No NV network	↑	-	↑	Drusenoid PED, RPD
5	OD	drusenoid PED	12	20/25	20/25	2× ranibizumab	7	-	↓	+	↓	Drusen
6	OS	drusenoid PED	36	20/40	20/63	none	persistent	-	↑	-	↑	St.p. macular hole, Drusen
7	OS	Drusen	6	20/70^a^	20/30	none	0	No NV network	↓	+	↔	Fibrovascular scar, Drusen

Clinical characteristics of the 7 included patients all diagnosed with AMD and presenting with a center-involving RPE defect with intact overlying photoreceptor layers and a minimum of follow up of 6 months. All included cases were treatment naïve without evidence of a choroidal neovascularization when diagnosed

*RPE* retinal pigment epithelium, *BCVA* best corrected visual acuity, *SRF* subretinal fluid, *BL* Baseline, *F/u* duration of follow up between initial presentation of RPE-defect and last visit, *FAF* fundus autofluorescence, *IRC* intraretinal cysts, *NV* neovascular, *Migration* migration of subretinal drusenoid/RPE material

^a^initial BCVA was performed prior to cataract surgery

The prior lesion preceding the RPE-defect was a drusenoid, avascular PED in the majority of the cases (*n* = 4) (Fig. 1; Table 1). Despite the absence of a definite choroidal neovascularization (CNV), 4 eyes with SRF received intravitreal anti-VEGF injections (Table 1). SRF (*n* = 5) persisted in 3 cases and the remaining 2 cases showed SRF resolution after 12 and 7 months, respectively. Resolution/persistence of SRF did not correspond to anti-vascular endothelial growth factor (VEGF) treatment (Table 1). Migration of RPE/drusenoid material right above the Bruch’s membrane with concomitant decrease of hypoautofluorescence and window defect was detectable in 4 cases (Figs. 1 and 2, Table 1). In 2 cases (case 2 and case 7) ingrowth was associated with visual acuity improvement. An increase of the RPE-defect size was apparent in the 3 remaining cases without tissue migration, all had persistent SRF and no evidence of migration of RPE/drusenoid material (Fig. 3). Figure 4 displays the SD-OCT images of all included patients.

## Representative cases

### Case 1

A patient presented with a drusenoid PED in 2010 (BCVA 20/20, Fig. 1). In 2011 there was spontaneous resolution of the PED and BCVA remained stable at 20/20. In April 2013 a spontaneous RPE-defect with overlying intact photoreceptors layers and SRF developed (BCVA 20/20). The patient received 3 monthly intravitreal bevacizumab injections without any visible change in the amount of SRF (BCVA 20/20). The treatment was stopped and the patient was observed. Spontaneous SRF resolution was noted November 2013. The intact EZ and ELM was now directly attached to the Bruch’s membrane (Fig. 1). Ingrowth of iso-and-hyperautofluorescent material was noted on FAF (Fig. 1, red arrows). Over the next 2 years, ingrowth and regeneration of RPE/drusenoid material with consecutive decrease of enhanced choroidal signaling, hypoautofluorescence and window defect was found. During this period BCVA remained stable at 20/20.

### Case 2

A patient presented with a circumscribed RPE-defect and a corresponding window defect on FA without evidence of retracted RPE in the left eye in 2010 with a BCVA of 20/160 (Fig. 2). The SD-OCT performed initially in April 2011 revealed the RPE-defect with enhanced choroidal signaling and intact ELM and EZ directly attached to the Bruch’s membrane (Fig. 2). Subretinal migration of hyperreflective material along the Bruch’s membrane with decrease of the hypo-FAF and the window defect in FA over the following 4 months was seen. During this period, the BCVA improved from 20/160 to 20/125 (Fig. 2). Throughout the next two years ELM and EZ remained intact, but the BCVA slowly dropped from 20/125 to 20/200.

### Case 3

In 2005 a patient noted a visual acuity decrease in the left eye. BCVA was 20/50 and soft, confluent drusen and a large, central RPE-defect were seen funduscopically (Fig. 3). FA showed early hyperfluorescence and late pooling without definite evidence of a CNV and without evidence of blockage due to retracted RPE (Fig. 3). The patient received a juxtascleral Retaane injection. BCVA remained unchanged. Over the next 2 years another Retaane injection and 3 intravitreal ranibizumab injections were administered. 2007 BCVA was 20/63 and SD-OCT revealed a central, large RPE-defect, SRF and preserved photoreceptor layers. Over the next 2 years the RPE-defect enlarged, and SRF persisted despite multiple ranibizumab injections. The ELM remained intact, but the EZ decreased in thickness and a reduction in photoreceptor outer segment shedding was noted (Fig. 3). BCVA was 20/80. 2013 intravitreal ranibizumab injections were stopped. The RPE-defect further enlarged and 2015 BCVA was 20/100. The OCT-A performed 2015 confirmed the absence of a CNV membrane in the left eye (Fig. 3).

## Discussion

We here describe a so far disregarded presentation of AMD with atypical RPE-defects not attributable to GA or RPE-tears with preserved overlying photoreceptor-layers. The majority had SRF which persisted or resolved independent to intravitreal treatment. This fact strengthens the assumption that not a CNV was the underlying cause of SRF but rather the local loss of the RPE and therefore the inability to pump ions and fluid out of the subretinal space. RPE-defect enlargement was associated with persistent SRF and lack of migration of subretinal RPE/drusenoid material, whereas migration of subretinal RPE/drusenoid material correlated with resolution/absence of SRF.

RPE-tears in the classical sense were first reported by Hoskin et al. and are characteristically found in patients with fibrovascular PED [14, 15]. They can occur spontaneously or may be related to various treatments [13, 15–18]. Contractile and hydrostatic forces inherent within the vascularized PED are described to be the most likely factors leading to the evolution of a RPE-tear, which usually involves the photoreceptor layers [15]. In general, RPE-tears in exudative AMD have a poor visual prognosis. Despite this fact, some cases with subfoveal RPE-tears and preserved visual function and retained photoreceptor layers were reported [11, 12, 19]. But our cases lacked characteristics of typical RPE-tears, which are typically found near the base of the fibrovascular PED with retracted RPE. There was also no evidence of exudative AMD, lacking the causative tractional forces of the CNV membrane. Further reasonable visual function and retained photoreceptors overlying the RPE-defect were evident and could be preserved over a long period of time.

GA may be the other well-known condition in AMD presenting with large RPE-defects. In contrast to our findings, RPE atrophy is associated with photoreceptor loss in GA. In fact, the loss of photoreceptors, in particular the loss of the rods and cone outer segments precedes RPE atrophy in GA and the area of photoreceptor loss may be much larger than the area of RPE atrophy [20–23]. Typical morphological patterns such as subsidence and thinning of the outer plexiform layer and loss of the ELM, the EZ- and the interdigitation zone is associated with RPE atrophy in GA [24].

The first report describing two patients with similar atypical RPE-defects was published 1987 [7]. These so called “blow-outs” of the RPE demonstrated intense leakage through the defects on FA. The resulting vision was 20/30 and 20/70, respectively. Similar to our cases and in contrast to characteristic RPE-tears, the RPE-defects were rather found at the dome of the detachment and not along the margins of the PED [7]. A case report published 1990 found two patients with spontaneous RPE-tears in long-standing serous RPE-detachments without evidence of a CNV who could keep a visual acuity of at least 20/40 over an observation period of 3 years after the rip had occurred [8]. During follow up, pigment migration along the tear edges was noted [8]. A RPE-tear in a patient with a mixed serous/drusenoid PED with intact photoreceptor layers and a visual acuity of 20/25 during the course of observation has recently been described [9]. Also in this patient, no definite evidence of a CNV was found [9]. Another report published 2014 showed spontaneous bilateral RPE-defects. After 2.5 years of follow-up the overlying photoreceptor layers were still intact and visual acuity were 6/9 and 6/6, respectively [25]. These examples all describe atypical, subfoveal RPE-defects with long-term survival of the overlying photoreceptors in patients with AMD and may depict the same disease process described here. A recent report described so called RPE-apertures [10]. Similar to our cases, well circumscribed discontinuity of the RPE without retracted RPE was found and avascular PEDs preceded respected lesions [10]. They enlarged homogeneously over time and focal hyperautofluoscent lesions were found prior to the onset [10]. Given the fact that 4 of our cases showed a drusenoid PED preceding the RPE-defect and hyperautofluosescent lesions, it is very likely that the RPE-apertures described, resemble the same disease process. However, none of the previously reported RPE apertures revealed migration of subretinal material.

Although 4 of our cases (case 2, 4 and 5, 6) had hyperautofluorescent lesions, an AMD related genesis, in particular a drusenoid PED rather than an underlying CSC or pattern dystrophy seems most likely as the age ranged between 71 and 87 years and they had large, soft drusen in the study as well as in the fellow eyes. However, a pattern dystrophy, CSC, Morbus Stargardt or a former Morbus Best may also be associated with such RPE alterations and cannot be excluded as possible underlying diseases.

Apparently photoreceptors seem capable to survive without normal RPE. A previous report already speculated that RPE may not obligatory be essential for the survival of photoreceptor-cells [8]. In AMD, rods seem more vulnerable, while cones seem to survive for longer periods also in the absence of outer or inner segments [23, 26, 27]. The reason for the earlier involvement of rods relative to cones in AMD is unknown, however it was speculated that the localized deficiency of retinoid, which is essential for photoreceptor survival, may be an underlying cause leading to impaired retinoid transfer from blood to the RPE due to debris accumulation [22]. Another explanation may be found in a study performed 2002, which demonstrated that within the neurosensory retina of non-human vertebrates there is an RPE-independent visual cycle for cone photopigment regeneration and day-light vision [28]. In accordance with those findings our long-term observation of preserved photoreceptor layers and reasonable visual function might uphold the hypothesis that (at least) the cone photopigment regeneration can occur independently from the RPE. The same previous study however also speculated that photoreceptors may be still apposed to some remaining RPE cells [8]. Unfortunately even in the OCT era, this question cannot be conclusively determined; additional image modalities such as polarization sensitive OCT or adaptive optics OCT may be able to further elaborate this question.

Beside the proposed mechanisms, other potential mode of actions may contribute to the long-term survival: Four of our cases showed migration of subretinal RPE/drusenoid material. The migration and recovery of pigmented tissue was initially noted and described 1988 in 4 patients after classic RPE-tears and it was speculated that it might resemble a repair mechanism [5] Bressler et al. also noticed, migration of pigmented material in both reported patients [8]. Caramoy et al. observed 2012 that patients with RPE-tears revealed migration and repopulation of RPE as shown in FAF and SD-OCT. However, this migrated RPE did not form a functional RPE-layer. They concluded that a small RPE-defect may be repaired by cell proliferation, but that respective RPE-cell proliferation is not sufficient in covering larger defects [29]. Mukai et al. recently described potential repair mechanisms in patients after RPE-tears and postulated 2 main modes of actions: The first mechanism included persistent SRF after the RPE-tear leading to thickened proliferative tissue at the affected area. The second mechanism was described to present with early and complete resolution of the SRF, the outer retina directly being attached to the Bruch’s membrane and attenuation of the normal hyperreflective band attributable to “normal” RPE [12]. The RPE/drusenoid material found in this report was also postulated to represent RPE-regeneration [12]. This assumption is strengthened by the fact that animal models have proven RPE-regeneration [8, 12, 30]. Accordingly, our cases showed SRF resolution and 2 cases had concomitant visual function improvement accompanied with the ingrowth of RPE/drusenoid material above the Bruch’s membrane, whereas in cases of persistent SRF no ingrowth was noted. Further, in cases of persistent SRF and no RPE ingrowth, enlargement of the RPE-defects has been observed. As the resolution of SRF was associated with the presence and ingrowth of the subretinal material it is likely that the tissue resembles RPE-regeneration, as previously suggested by abovementioned reports with the capability of normal RPE to actively pump ions and fluid out of the subretinal space. RPE regeneration may also explain the survival of the photoreceptors in patients with respective ingrowth.

Limitation of this study includes its retrospective design and the small number of patients which is mainly due to the fact that this finding is rather rare in contrast to GA.

## Conclusion

To summarize, we here present a so far disregarded presentation of AMD with atypical RPE-defects and preserved overlying photoreceptor-layers over a mean of 4 years. Affected eyes retained reasonable vision without any evidence of CNV. Repair mechanisms such as ingrowth of RPE/drusenoid material, as well as a RPE-independent visual cycle for cone photopigment within the neurosensory retina may contribute to the favorable long-term results.

## Abbreviations

AMD: Age-related macular degeneration
BCVA: Best corrected visual acuity
CNV: Choroidal neovascularization
CSC: Central serous chorioretinopathy
ELM: External limiting membrane
EZ: Ellipsoid zone
FA: Fluorescein angiography
FAF: Fundus autofluorescence
GA: Geographic atrophy
ICG: Indocyanine angiography
OCT: Optical coherence tomography
PED: Pigment epithelium detachment
PR: Photoreceptor
RPE: Retinal pigment epithelium
SD-OCT: Spectral domain optic coherence tomography
SRF: Subretinal fluid
VEGF: Vascular endothelial growth factor
